# Expression of Key Ion Transporters in the Gill and Esophageal-Gastrointestinal Tract of Euryhaline Mozambique Tilapia *Oreochromis mossambicus* Acclimated to Fresh Water, Seawater and Hypersaline Water

**DOI:** 10.1371/journal.pone.0087591

**Published:** 2014-01-31

**Authors:** Zhengjun Li, Eei Yin Lui, Jonathan M. Wilson, Yuen Kwong Ip, Qingsong Lin, Toong Jin Lam, Siew Hong Lam

**Affiliations:** 1 NUS Environmental Research Institute, National University of Singapore, Republic of Singapore; 2 Department of Biological Science, National University of Singapore, Republic of Singapore; 3 Ecofisiologia, Centre for Interdisciplinar Marine and Environmental Research, University of Porto, Porto, Portugal; Universitat de Barcelona, Spain

## Abstract

The ability of euryhaline Mozambique tilapia to tolerate extreme environmental salinities makes it an excellent model for investigating iono-regulation. This study aimed to characterize and fill important information gap of the expression levels of key ion transporters for Na^+^ and Cl^−^ in the gill and esophageal-gastrointestinal tract of Mozambique tilapia acclimated to freshwater (0 ppt), seawater (30 ppt) and hypersaline (70 ppt) environments. Among the seven genes studied, it was found that *nkcc2*, *nkcc1a*, *cftr*, *nka-α1* and *nka-α3*, were more responsive to salinity challenge than *nkcc1b* and *ncc* within the investigated tissues. The *ncc* expression was restricted to gills of freshwater-acclimated fish while *nkcc2* expression was restricted to intestinal segments irrespective of salinity challenge. Among the tissues investigated, gill and posterior intestine were found to be highly responsive to salinity changes, followed by anterior and middle intestine. Both esophagus and stomach displayed significant up-regulation of *nka-α1* and *nka-α3*, but not *nkcc* isoforms and *cftr*, in hypersaline-acclimated fish suggesting a response to hypersalinity challenge and involvement of other forms of transporters in iono-regulation. Changes in gene expression levels were partly corroborated by immunohistochemical localization of transport proteins. Apical expression of Ncc was found in Nka-immunoreactive cells in freshwater-acclimated gills while Nkcc co-localized with Nka-immunoreactive cells expressing Cftr apically in seawater- and hypersaline-acclimated gills. In the intestine, Nkcc-stained apical brush border was found in Nka-immunoreactive cells at greater levels under hypersaline conditions. These findings provided new insights into the responsiveness of these genes and tissues under hypersalinity challenge, specifically the posterior intestine being vital for salt absorption and iono-osmoregulation in the Mozambique tilapia; its ability to survive in hypersalinity may be in part related to its ability to up-regulate key ion transporters in the posterior intestine. The findings pave the way for future iono-regulatory studies on the Mozambique tilapia esophageal-gastrointestinal tract.

## Introduction

The euryhaline teleost, *Oreochromis mossambicus* also known as the Mozambique tilapia, can be acclimated to extreme environmental salinities ranging from freshwater (FW), seawater (SW) to hypersaline water (HSW) up to four-fold the salinity of SW [1], [2]. In order to maintain body fluid homeostasis, the tilapia has to cope with the iono-osmoregulatory challenges exerted by these extreme environmental salinities by dynamically regulating ion and water balance. In hypo-osmotic FW environments, passive osmotic water gain needs to be minimized and excess water removed from the body, while the loss of salt needs to be minimized if not replaced by active sequestering from the environment. In hyper-osmotic SW environments, osmotic water loss needs to be reduced if not replaced by ingestion of SW and the excess salt gain needs to be actively excreted from the body. These iono-osmoregulatory challenges escalate dramatically as environmental salinity increases beyond SW into hypersaline levels where only few teleost species, including several tilapias, have evolved the extraordinary capability to iono-osmoregulate in HSW environments [3]–[5].

Studies have been conducted to investigate the iono-osmoregulatory mechanisms of gills in tilapia. Most studies have focused mainly on isoforms of key ion transporters such as Na^+^/K^+^-ATPase (Nka), Na^+^∶K^+^∶2Cl^−^ cotransporter (Nkcc), Na^+^∶Cl^−^ cotransporter (Ncc), cystic fibrosis transmembrane regulator (Cftr) Cl^−^ channel and several ion exchangers within the gills by comparing their expression at both gene and/or protein levels in FW and SW environments [6]–[9]. However, there is little information with regards to the expression levels of these ion transporters in the tilapia gill under HSW environments and even much less is known in the esophageal-gastrointestinal (EGI) tract of tilapia acclimated to different environmental salinities. The quantitative changes in gene expression levels of these ion transporters in gills acclimated to HSW has yet to be determined, although biochemical and physiological changes in gills had been investigated [10]–[12], while quantitative morphological changes of gill ionocytes in tilapia acclimated to HSW had been studied using ultrastructural [6] and immunohistochemical [9] approaches.

As for the tilapia EGI tract, there has been limited information with regards to its role in iono-osmoregulation and the expression of these key transporters along the different segments of the EGI tract in FW, SW and HSW acclimated fish. The EGI tract plays an important osmoregulatory role in compensating water loss in SW environment by selective salt and water uptake from ingested SW [13]. However, despite its crucial iono-osmoregulatory role, the EGI tract in teleosts has generally received much lesser attention than the gill in iono-osmoregulation studies [14]. In tilapia, the EGI tract, in part or in whole, has been studied in salinity challenge experiments with regards to glucose transport [15], [16], total carbon dioxide concentration [17], endocrine responses [18]–[20], and cell proliferation-apoptosis [21]. Gene expression of ion transporters has been detected for ‘intestine’ as a whole organ in tilapia acclimated in FW and SW [7] but not in their morpho-functional segments. This warrants the present study because the tilapia EGI tract is known to be morpho-functionally divided [22], hence we hypothesize that the different segments of the EGI tract would display different iono-osmoregulatory levels.

This study aimed to fill in the knowledge gap on the expression of selected key ion transporters involved in the ion regulation of the main ions Na^+^ and Cl^−^ in gill and EGI tract of the euryhaline Mozambique tilapia model acclimated in FW-, SW- and HSW-environments. We have cloned and quantified the gene expression of seven major ion transporters (*nkcc1a*, *nkcc1b*, *nkcc2*, *ncc*, *cftr*, and *nka-α1*, and *nka-α3*) in gills and EGI tract of tilapia acclimated in FW (0 ppt), SW (30 ppt) and HSW (up to 70 ppt). These genes were selected as they are the key ion transporters known to be critical for Na^+^ and Cl^−^ ion regulation in FW and SW environments. The expression profiles of these genes were assessed in gills and five segments of EGI tract including the esophagus, the stomach, the anterior intestine (AI), the middle intestine (MI), and the posterior intestine (PI) to gain insight into the iono-osmoregulatory roles of these regions under hypersalinity stress. In addition, we have also performed immunohistochemical staining for localization of Nkaα, Nkcc/Ncc, and Cftr in gill and anterior and posterior intestine. We observed gene expression profiles that were similar, as well as those that were distinct from each other in the gills and the EGI tract of fish acclimatized to different salinities. Based on the known functions of these transporters and their localizations in epithelial membrane, these findings provide new insights into Na^+^ and Cl^−^ ion regulation in gills and along the EGI tract of tilapia in FW, SW and HSW-environments.

## Materials and Methods

### Ethics statement

Animal procedures adopted in this study were approved by the Institutional Animal Care and Use Committee of the National University of Singapore (IACUC 098/10).

### Fish and experimental protocol

Mozambique tilapia (*Oreochromis mossambicus*) measuring 10–15 cm in total length were purchased from a local commercial fish farm and were maintained in 200-L tanks with recirculating dechlorinated tap water (FW) with gentle aeration at 25–26°C under 12 h light∶ 12 h dark photoperiod for at least 2 weeks before experiments. Fish were fed twice daily with commercial fish food (Hikari cichlid bio-gold, Kyorin food Ind. Ltd.) until one day before sampling. Fish were randomly assigned into 3 groups. Group I was maintained in FW as control group. Group II and III were acclimated stepwise to natural seawater (SW, 30 ppt) over five transfers (10 ppt, 15 ppt, 20 ppt, 25 ppt, 30 ppt), with two days to allow for acclimation at each stage of increasing salinity. Group II was then maintained for about three weeks in SW until sampling. Meanwhile, group III tilapia were acclimated stepwise from SW to hypersaline water (HSW, 70 ppt) over eight transfers (35 ppt, 40 ppt, 45 ppt, 50 ppt, 55 ppt, 60 ppt, 65 ppt, 70 ppt), with two days allowed for acclimation at each stage, and finally maintained at 70 ppt for four days before sampling. The entire step-wise acclimation from SW to HSW took about 3 weeks and thereafter fish from FW, SW and HSW were sampled on the same day. Water with salinities below 30 ppt were prepared by mixing dechlorinated tap water with natural SW, while water above 30 ppt to HSW were prepared by adding sodium chloride (Schedelco, Malaysia) to SW. Salinity was determined using a light refractometer.

Fish were anaesthesized with 0.1% (v/v) 2-phenoxyethanol (Sigma-aldrich, USA) before tissue sampling. Gill filaments were excised, while the esophagus, stomach, anterior intestine (AI), middle intestine (MI), and posterior intestine (PI) were dissected and placed in RNAlater (Ambion, USA) before cutting the tissues into smaller pieces. The cut tissue samples were then immediately snap-frozen in liquid nitrogen and stored at −80°C until used. For histological analysis, tissues were fixed in 4% (w/v) paraformaldehyde in phosphate-buffered saline for immunohistochemical staining.

### RNA extraction and cDNA synthesis

Total RNA isolation from tilapia gill or various segments of EGI tract was performed using TRIzol reagent (Invitrogen, USA) according to the manufacturer's protocols. RNA purity and quantity was measured using Nanodrop ND-2000 spectrophotometer (Thermo Fisher, USA). RNA were treated with DNase I, amplification grade (Invitrogen, USA) to remove any contaminating genomic DNA. First-strand cDNA was synthesized by reverse-transcription from 3 µg of total RNA using SuperScript II reverse transcriptase (Invitrogen, USA) with oligo(dT)_20_.

### Cloning and sequencing of construct

The full-length cDNAs encoding Nkcc1a (GenBank accession No. AY513737; 3456 bp), Nkcc1b (AY513738; 3288 bp), Nkcc2 (AY513739; 3126 bp) and Ncc (EU518934; 3003 bp) and partial sequences of Nka-α1 (U82549; 837 bp from nucleotide position 142 to 978 bp), Nka-α3 (AF109409; 1020 bp; from nucleotide position 205 to 1224 bp), Cftr (AB601825; 1206 bp; from nucleotide position 1 to 1206 bp) have been PCR amplified from first-strand cDNA samples. The amplified genes were digested with the restriction enzyme Xba I and Xho I and then ligated to the similarly digested vector pBluescript II KS(-) (Agilent Technologies; Palo Alto, CA, USA). These cloned genes were sequenced using ABI PRISM 377 DNA Sequencer (Applied Biosystems, Carlsbad, CA, USA) and have been confirmed by alignment with their respective sequences downloaded from NCBI database. The primers used for these constructs are listed in supporting information **Table S1**.

### Quantitative real-time PCR

Absolute quantification real-time PCR was carried out as described previously [23] to determine the mRNA levels of tilapia *nkcc1a*, *nkcc1b*, *nkcc2*, *ncc*, *nka-α1*, *nka-α3* and *cftr*. Real-time PCR was performed on a StepOnePlus™ Real-Time PCR system (Applied Biosystems, USA) in a 10 µl reaction volume using 50 ng cDNA, 200 nM forward and reverse primers and 5 µl of Express SYBR GreenER qPCR supermix with premixed ROX (Invitrogen, USA). The concentration of the cDNA samples and plasmid cDNA constructs were determined using Nanodrop ND-2000 spectrophotometer (Thermo Fisher, USA). The copy numbers of the plasmid cDNA constructs were calculated according to the molecular weight of the plasmid (average value = 660/bp) and then converted into the copy numbers using Avogadro's number (1 mol = 6.022×10^23^ molecules) based on their respective concentrations. Serial ten-fold dilutions (from 10^7^ to 10^1^ copies/µl) of the plasmid cDNA constructs were run in triplicate to generate standard curves. Each of the tissue sampled from 8 individual fish for each treatment condition (FW, SW, and HSW) was used individually for real-time PCR quantification for each gene (n = 8). Cycling conditions were 95°C for 20 s followed by 40 cycles of 95°C for 3 s and 60°C for 30 s. Amplification was followed by a melting curve analysis to confirm the specificity of the PCR reaction. Standard curves were obtained from plotting C_T_ on the y-axis and the natural log of concentration (copies/µl) on the x-axis. The unknown quantity of transcript in a sample was determined from the linear regression line derived from the standard curve and the copy numbers per 50 ng cDNA were determined. The primers used for quantitative real-time PCR are listed in supporting information **Table S2**.

### Immunohistochemistry

Gill and gastro-intestinal tissues were excised and fixed in 4% paraformaldehyde (PFA) in phosphate buffered saline (PBS) (pH 7.4) for 24 h at 4°C, processed for paraffin embedding, and sectioned as described by Wilson et al. [24]. Following dewaxing and rehydrations, antigen retrieval was performed using 0.05% citraconic anhydride (pH 7.3) for 30 min at 100°C [25] and 1%SDS/PBS for 5 min [26] and thoroughly rinsed. Sections were then blocked with 5% normal goat serum (NGS)/1% bovine serum albumin (BSA)/0.05% Tween 20-phosphate buffered saline (TPBS; pH 7.4) for 15 min and incubated with either mouse monoclonal anti-Cftr antibody (Clone 24-1; R&D systems) or monoclonal anti-Nkcc/Ncc antibody (clone T4; DSHB [27]) with a rabbit anti-Na^+^/K^+^ ATPase α subunit polyclonal antibody (1∶500 [24]) diluted in 1∶200, 1∶100 and 1∶500, respectively in 1% BSA/TPBS (0.05% Tween-20/PBS, pH 7.4)/0.05% sodium azide overnight at 4°C in humidity chambers. Negative control incubations were performed simultaneously under the same conditions, using isotyped hybridoma culture supernatant (clone J3), and either preimmune rabbit serum or antibody pre-absorbed with excess peptide (pre-absorbed overnight at 4°C on an orbital shaker) equivalently diluted as the primary antibodies. Secondary incubations were performed with goat anti-mouse Alexa Fluor 488 and goat anti-rabbit Alexa Fluor 568 conjugated secondary antibodies (Invitrogen S.A., Barcelona, Spain) diluted 1∶400 in 1%BSA/TPBS, for 1 h at 37°C. Nuclei were counterstained with 4′,6-Diamidino-2-phenylindole (DAPI) and coverslips were mounted with 1∶1 glycerol : PBS, pH 7.5 and observed on an epifluorescence microscope (Leica Microsystems DM6000 B, Germany). Images of fluorescent staining were captured with a Leica DFX340 camera, along with the corresponding differential interference contrast (DIC) image. Plates were assembled using Adobe Photoshop CS3 software, and images enhanced while maintaining the integrity of the data.

Tissue fluorescent staining was quantified using image analysis software (SigmaScanPro v.5 SPSS, Chicago IL USA). The use of image analysis software for quantifying immunofluorescent signal in tissue section has been reported previously [28]. Images for a given antibody were collected under identical capture conditions from randomly selected non-contiguous fields. The results are expressed as a ratio of the luminosity of Alexa 488 (Nka) or Alexa 568 (Nkcc/Ncc) to DAPI (nuclei) staining within a total individual field of 0.307 mm^2^; the DAPI (nuclei) staining is used as a proxy for tissue area to correct for differences between fields. This is subsequently referred as ‘normalized luminosity index’. A total of 90 images were analyzed per tissue (gill, anterior intestine and posterior intestine).

### Statistical analysis

The gene expression data and immunohistochemical data were analyzed statistically by one-way analysis of variance (ANOVA) followed by *post-hoc* Duncan's multiple range test using SPSS Statistics (IBM, USA). A value of *P*<0.05 was considered to be statistically significant in the analysis.

## Results

### Changes in gene expression levels of selected key ion transporters in gills and EGI tract of FW-, SW- and HSW-acclimated fish

**Figure 1:**
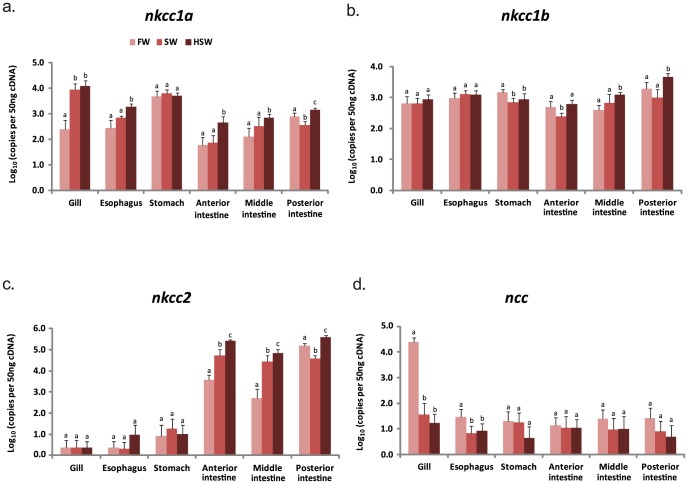
Gene expression of *sodium (potassium) chloride* (*nkcc/ncc*) *cotransporters*. Expression levels of *nkcc1a* (a), *nkcc1b* (b), *nkcc2* (c), *ncc* (d), in the gills and EGI tract of tilapia acclimated to freshwater (FW), seawater (SW) and hypersaline water (HSW). Each histogram bar represents the mean ± s.d. of the expression levels (log_10_ copies of transcript per 50 ng cDNA). Expression levels labeled with different lowercase alphabets are significantly different (one-way ANOVA followed by Duncan's post-hoc test; *P*<0.05) within the same tissue.

The absolute mRNA expression levels of *nkcc1a*, *nkcc1b*, *nkcc2*, *ncc*, *cftr*, and *nka-α1* and *nka-α3* were determined in the gills and five different segments of EGI tract including the esophagus, stomach, AI, MI, and PI in fish acclimated to FW, SW (30 ppt), and HSW (70 ppt) environments. The expression of each gene in each tissue were compared between FW-, SW- and HSW-acclimated fish to determine if their expression levels within each tissue type were affected by salinity challenge (**Figure 1**). The relative expression fold-change was approximated from the expression levels.

### Gene expression of *nkcc1a* and *nkcc1b*

In the gill, the expression of *nkcc1a* was significantly (*P*<0.05) up-regulated in SW- and HSW-acclimated fish when compared to FW-acclimated fish (**Figure 1a**). In the EGI tract of SW-acclimated fish, *nkcc1a* expression significantly (*P*<0.05) decreased 2-fold in the PI but did not show significant (*P*>0.05) change albeit slight increase in other segments (**Figure 1a**). However, upon greater salinity challenge in HSW-acclimated fish, *nkcc1a* was significantly up-regulated in the esophagus, and all the three intestinal segments (**Figure 1a**). The mean expression of *nkcc1a* significantly (*P*<0.05) increased 35- and 50-fold in the respective gills of SW- and HSW-acclimated fish when compared to the gill of FW-acclimated fish. Interestingly, the expression of *nkcc1a* in FW-acclimated fish was most abundant in the stomach (about 6 to 40-fold higher than other tissues) and remained at similar levels in the SW- and HSW-acclimated fish, suggesting that its high expression in the stomach does not respond to salinity challenge (**Figure 1a**). The findings indicate that *nkcc1a* is highly responsive to salinity challenge in the gills and moderately responsive in the esophagus and intestine.

No significant (*P*>0.05) changes of branchial *nkcc1b* expression were detected in fish acclimated SW or HSW when compared to FW (**Figure 1b**) suggesting that *nkcc1b*, unlike *nkcc1a*, is not responsive to salinity challenge in the gill. In the EGI tract, however, expression of *nkcc1b* was significantly (*P*<0.05) down-regulated in the stomach of SW- and HSW-acclimated fish, as well as in the AI of SW-acclimated fish, but was significantly (*P*<0.05) up-regulated in the MI and the PI of HSW-acclimated fish (**Figure 1b**). Among the intestinal segments, *nkcc1b* transcript was most abundant in the PI irrespective of salinity (**Figure 1b**). The findings indicate that *nkcc1b* is moderately responsive to salinity challenge in the EGI tract.

### Gene expression of *nkcc2*

The expression of *nkcc2* was detectable at very low levels in the tilapia gills, esophagus and stomach, but was abundantly expressed in the intestinal segments of FW-, SW- and HSW-acclimated fish (**Figure 1c**). In FW-acclimated fish, *nkcc2* expression was detected most abundantly in the PI, about 40-fold and 300-fold higher than the AI and MI, respectively. In SW-acclimated fish, the expression of *nkcc2* increased significantly (*P*<0.05) to 14- and 51-fold in the AI and MI, respectively, but was significantly (*P*<0.05) down-regulated 4-fold in the PI, when compared to FW-acclimated fish (**Figure 1c**). When challenged with greater salinity, *nkcc2* expression was significantly (*P*<0.05) up-regulated 70-, 135- and 2.5-fold in the respective AI, MI and PI, of HSW-acclimated fish when compared with the FW-acclimated fish (**Figure 1c**). These findings indicate that *nkcc2* is abundantly expressed in the EGI tract and is highly responsive to salinity challenge.

### Gene expression of *ncc*

The highest abundance of *ncc* transcript was detected in the gills of FW-acclimated fish but was significantly (*P*<0.05) down-regulated 555- and 1200-fold in SW and HSW-acclimated fish, respectively (**Figure 1d**). Low levels of *ncc* transcripts were also detected in the esophagus of FW-acclimated fish which was again significantly (*P*<0.05) down-regulated in SW- and HSW-acclimated fish (**Figure 1d**). The expression of *ncc* in the EGI tract remained very low with no significant (*P*>0.05) changes in fish acclimated to all three environments suggesting that it is not responsive to salinity challenge in the stomach and intestine. The marked down-regulation of branchial *ncc* expression in SW and HSW environments indicates that the expression of *ncc* is sensitive to salinity challenge in the gill and the encoded ion transporter is mainly required in low ionic, hypo-osmotic FW environment.

### Gene expression of *cftr*

**Figure 2:**
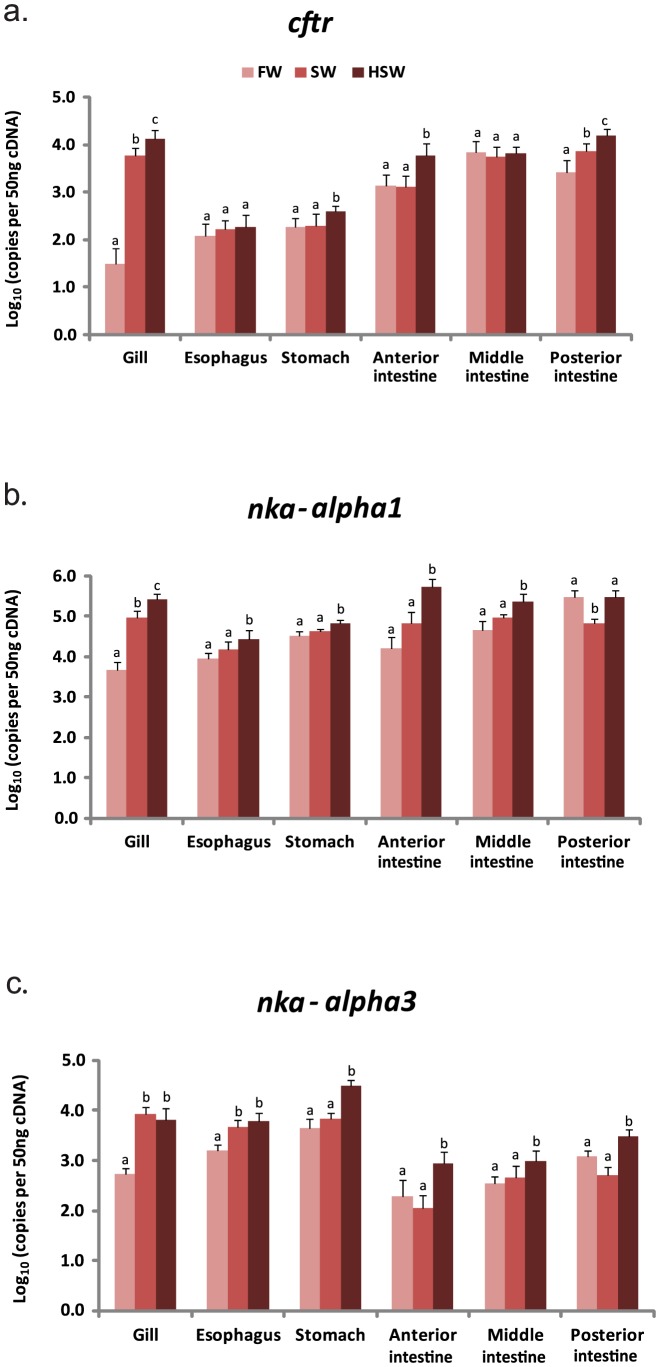
Gene expression of *cystic fibrosis transmembrane conductance regulator* (*cftr*) and *sodium potassium ATPases* (*nka*). Expression levels of *cftr* (a), *nka*-α1 (b), *nka*-α3 (c) in gills and EGI tract of tilapia acclimated to freshwater (FW), seawater (SW) and hypersaline water (HSW). Each histogram bar represents the mean ± s.d. of the expression levels (log_10_ copies of transcript per 50 ng cDNA). Expression levels labeled with different lowercase alphabets are significantly different (one-way ANOVA followed by Duncan's post-hoc test; *P*<0.05) within the same tissue.

The expression of *cftr* was low in the FW- acclimated gill but was significantly (*P*<0.05) up-regulated by 190- and 435–fold in the gill acclimated to SW and HSW environments, respectively (**Figure 2a**). In the EGI tract, higher expression of *cftr* was consistently observed in the intestinal segments than the esophagus and the stomach regardless of salinity (**Figure 2a**). When compared to FW-acclimated fish, *cftr* expression was significantly (*P*<0.05) up-regulated 2-fold and 6-fold in the PI of SW- and HSW-acclimated fish, respectively, as well as significantly (*P*<0.05) up-regulated 4-fold in the AI and 2-fold in the stomach of HSW-acclimated fish (**Figure 2a**). The findings indicate that *cftr* is highly responsive to salinity challenge in the gills and moderately responsive in the EGI tract, particularly in the AI and the PI.

### Gene expression of *nka-α1* and *nka-α3*

The expression of *nka-α1* and *nka-α3* were abundant in all the tissues investigated, and the expression level of *nka-α1* is about 10-fold higher than *nka-α3* in the corresponding tissues and environments (**Figure 2b and 2c**). In the gills, *nka-α1* expression was significantly (*P*<0.05) up-regulated 20- and 50-fold in the SW- and HSW-acclimated fish, respectively, when compared to the FW-acclimated fish (**Figure 2b**). Likewise, branchial *nka-α3* significantly (*P*<0.05) increased 17- and 13-fold in the SW- and HSW-acclimated fish, respectively, when compared to the FW-acclimated fish (**Figure 2c**). In the EGI tract, the expression of *nka-α1* was significantly (*P*<0.05) up-regulated in the esophagus (3-fold), stomach (2-fold), AI (33-fold) and MI (5-fold) of HSW-acclimated fish, but significantly (*P*<0.05) down-regulated in the PI (4-fold) of SW-acclimated fish, when compared to the FW-acclimated fish (**Figure 2b**). As for *nka-α3*, its expression was significantly (*P*<0.05) up-regulated in the esophagus (3-fold) of SW-acclimated fish, as well as in the esophagus (4-fold), stomach (7-fold), AI (4.5-fold), MI (3-fold) and PI (2.5 fold) of HSW-acclimated fish, when compared to FW-acclimated fish (Figure 2c). The findings suggest that both *nka-α1* and *nka-α3* are highly responsive to salinity challenge in the gill and moderately responsive in the EGI tract.

### Gene expression profiles of selected key ion transporters in gills and EGI tract of FW-, SW- and HSW-acclimated fish

**Figure 3:**
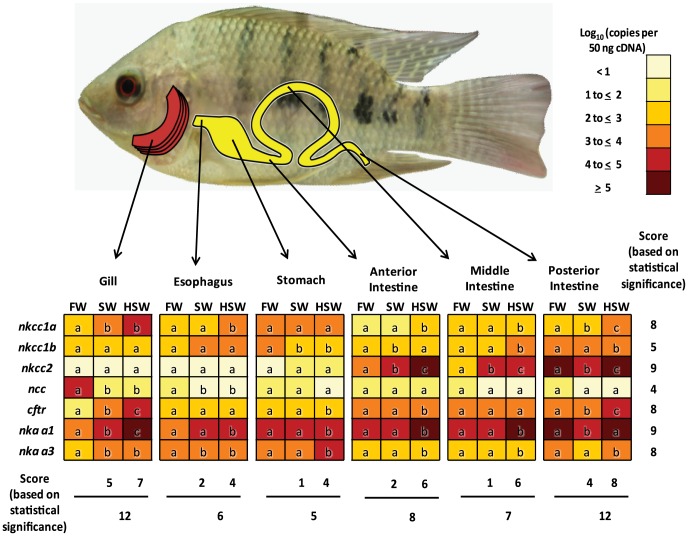
Gene expression profiles in gills and gastro-intestinal tract of tilapia acclimated different salinities. Heatmap representing quantity (log_10_ copies per 50 ng cDNA) of *nkcc1a*, *nkcc1b*, *nkcc2*, *ncc*, *cftr*, *nka-α1*, and *nka-α3* in the gills and EGI tract segments, including the esophagus, stomach, anterior intestine (AI), middle intestine (MI), and posterior intestine (IG) of tilapia acclimated freshwater (FW), seawater (SW) and hypersaline water (HSW). Different alphabets indicates significant (*P*<0.05) differential gene expression between the salinity conditions (refer to text for the scoring system) within a tissue.

To gain overall perspective and derive significance of the expression profiles for the seven genes within the six tissues with regards to salinity challenge, we further condensed our expression data into a heatmap (**Figure 3**) and transferred the statistical significance (*P*<0.05) represented by different alphabets from **Figure 1** and **Figure 2** into **Figure 3**. We then scored for the number of significant (*P*<0.05) differences. In the case of two-group difference, e.g. if a gene expression in HSW and/or SW is significantly (*P*<0.05) different when compared with FW only, an alphabet ‘b’ is assigned in the respective HSW and/or SW cells while FW is assigned alphabet ‘a’. In the case of three-group difference i.e. if a gene expression in HSW is significantly different when compared to FW and SW, where SW is also significantly different from FW, alphabet ‘b’ is assigned in the SW cell and alphabet ‘c’ is assigned in the HSW cell while FW is assigned alphabet ‘a’. Alphabets ‘a’, ‘b’ and ‘c’ are scored as 0, 1 count and 2 counts of significant difference, respectively. By totaling the number of significant differential expression for each tissue and each gene, we were able to determine the genes that were deregulated most frequently and the tissues that had the highest number of deregulated genes in response to salinity challenge.

Among the seven genes investigated, *nkcc2*, *nkcc1a*, *cftr*, *nka-α1* and *nka-α3*, have the highest score (8–9) for significant differential expression when compared to *ncc* and *nkcc1b* which respectively have 4 and 5 significant differential expression in the tissues that were investigated (**Figure 3**). Therefore, *nkcc2*, *nkcc1a*, *cftr*, *nka-α1* and *nka-α3*, were genes that were more responsive to salinity challenge in these tissues when compared to *nkcc1b* and *ncc*.

Among the tissues investigated with regards to the expression of these genes, the gill and PI both scored 12 significant differential expression followed by 8 for AI and 7 for MI, while esophagus and stomach scored 6 and 5 significant differential expression, respectively (**Figure 3**). In the gill of FW-acclimated fish, *ncc* and *nka*-*α1* were highly expressed while the transcripts for *nkcc2* and *cftr* were almost non-detectable.

In the gills of SW-acclimated fish, the *ncc* expression was significantly down-regulated to very low levels while transcripts for *nka-α1*, *nka-α3*, *cftr* and *nkcc1a* were increased significantly. Similar profiles were observed in the gill of HSW-acclimated fish, with significantly higher branchial *nka-α1* and *cftr* expressions than SW-acclimated fish. In the PI of FW-acclimated fish, *nkcc2* and *nka-α1* transcripts were highly abundant followed by *nkcc1b*, *cftr* and *nka-α3* transcripts which were moderately abundant. In the PI of SW-acclimated fish, the expression of *cftr* was up-regulated while *nkcc1a*, *nkcc2* and *nka-α1* were down-regulated when compared to FW-acclimated fish. In HSW environment, the PI was highly responsive with the expression of *nkcc1a*, *nkcc1b*, *nkcc2* and *cftr* significantly up-regulated when compared to SW- and FW-acclimated fish.

Both AI and MI shared similar expression profiles with significant up-regulation of *nkcc2* in SW-acclimated fish, and significant up-regulation of *nkcc1a*, *nkcc2*, *nka-α1* and *nka-α3* in HSW-acclimated fish. In the esophagus, *ncc* was significantly down-regulated and *nka-α3* was significantly up-regulated in SW- and HSW-acclimated fish, while *nka-α1* and *nkcc1a* were significantly up-regulated in the HSW-acclimated fish when compared to FW-acclimated fish. In stomach, only *nka*-α1, *nka*-α3 and *cftr* were significantly increased in HSW-acclimated fish when compared to FW-acclimated fish. Taken together, the gill and PI followed by AI and MI were most responsive to salinity challenge while esophagus and stomach was the least responsive hence highlighting the relative importance of these genes in their iono-regulatory roles within these tissues.

### Immunohistochemical staining of selected key ion transporters in gills and intestine of FW-, SW- and HSW-acclimated fish

**Figure 4:**
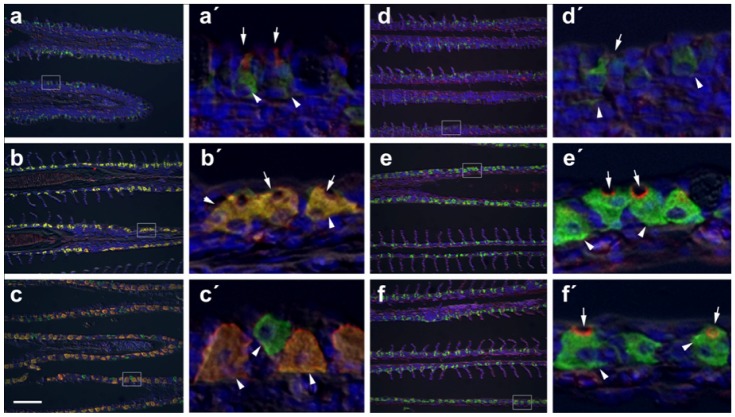
Immunohistochemical localization of transporters in gills of tilapia acclimated to different salinities. Representative micrographs of immunohistolocalization of Nka (green) with either Nkcc/Ncc (a–c; red) or Cftr (d–f; red) in the gills of tilapia acclimated to FW (a,d), SW (b,e) or HSW (c,f). Co-localization of red and green fluorochromes results in yellow-orange staining. Higher magnification (10×) of boxed areas in (a–f) correspond to panels (a′–f′). Sections are counter stained with the nuclear stain DAPI and overlaid with the DIC image for tissue orientation. Arrows indicate apical staining and arrowheads tubular system (basolateral) staining. Scale bar 100 µm (a–f), 10 µm (a′–c′).

**Figure 5:**
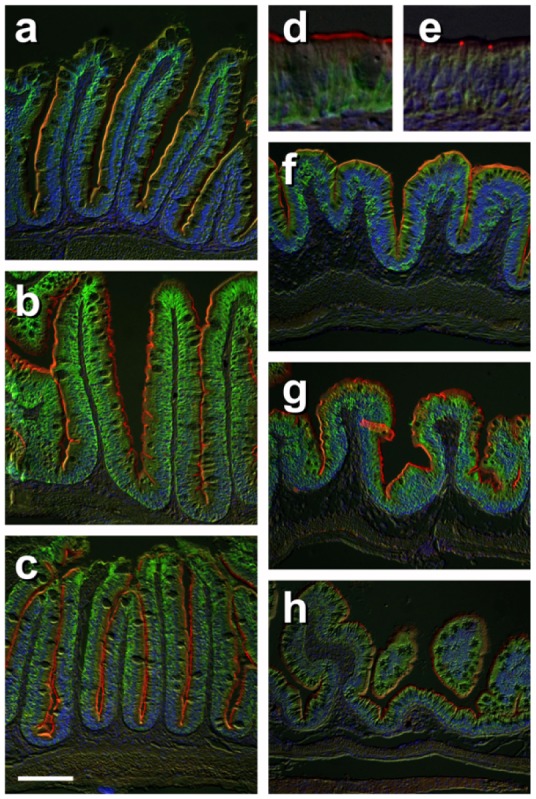
Immunohistochemical localization of transporters in anterior and posterior intestine of tilapia acclimated to different salinities. Representative micrographs of immunolocalization of Nka (green) with Nkcc/Ncc (red) (a–d, f–h) from FW (a, f), SW (b, g) and HSW (c, h) acclimated tilapia. (d) A representative higher magnification micrograph of Nkcc/Ncc staining of the brush border of enterocytes with basolateral Nka staining from the anterior intestine of SW-acclimated fish. (e) Apical Cftr (red) double labeling with Nka (green) in the anterior intestine of a FW-acclimated fish. Panels (a–e) are sections of anterior intestine (AI) while panels (f–h) are sections of posterior intestine (PI). Sections are counter stained with the nuclear stain DAPI and overlaid with the DIC image for tissue orientation. Scale bar 100 µm (a–c, f–h); 25 µm (d,e).

We further performed immunohistochemical staining on the gill, AI and PI as representative tissues that were more responsive to salinity challenge (**Figure 4** and **5**). The tilapia gill has strong Nka immunoreactive cells located primarily in the filament epithelium in an apparent cytosolic location (**Figure 4**). This pattern is related to the extensive tubular system which is in continuity with the basolateral membrane of these cells [29]. In FW-acclimated fish, Ncc is localized apically in a subpopulation of these cells (**Figure 4a** and **4a′**). This apical staining of Ncc is not present in the gills of SW- or HSW-acclimated fish. Under increasing salinity, Nkcc is co-localized with Nka associated with the basolateral tubular system and the intensity of Nkcc staining increased with salinity (**Figure 4a–4c** and **4a′–4c′**). Likewise, Nka-Nkcc immunoreactive cells in the gills appeared to have increased noticeably in size and numbers with increasing salinity. Indeed the normalized luminosity indices for Nkcc/Ncc and Nka immunostaining were significantly (*P*<0.005) higher in the gills of SW- and HSW-acclimated fish when compared to FW-acclimated fish (**Table 1**). Interestingly, a small but distinct population of Nka immunoreactive cells which lacked Nkcc staining but are closely associated with Nka-Nkcc immunoreactive cells became more apparent in the gill of HSW-acclimated fish (**Figure 4c** and **4c′**). Cftr is localized to the apical crypt membrane of Nka-immunoreactive cells in the gills of SW- and HSW-acclimated fish but not in FW-acclimated fish (**Figure 4d–f** and **4d′–f′**).

**Table 1:** Normalized luminosity indices of Nkcc/Ncc and Nka immunostaining in the gills, anterior intestine (AI) and posterior intestine (PI) of Mozambique tilapia.

Tissues and Conditions (number of replicates)	Normalized Luminosity Index* for Nkcc/Ncc	Normalized Luminosity Index* for Nka
**Gill**		
Freshwater (FW; *n* = 4)	0.811±0.016^a^	0.726±0.013^a^
Seawater (SW; *n* = 3)	0.922±0.010^b^	0.824±0.010^b^
Hypersaline water (HSW; *n* = 3)	0.996±0.024^c^	0.871±0.033^b^
*P-value* **	<0.001	<0.005
**Anterior Intestine (AI)**		
Freshwater (FW; *n* = 4)	0.803+0.037^a^	0.807+0.033^a^
Seawater (SW; *n* = 3)	0.975+0.063^b^	0.908+0.049^a^
Hypersaline water (HSW; *n* = 3)	1.013+0.056^b^	1.006+0.066^a^
*P-value*	<0.05	0.061
**Posterior Intestine (PI)**		
Freshwater (FW; *n* = 4)	0.853+0.014^a^	0.842+0.018^a^
Seawater (SW; *n* = 3)	0.956+0.075^ab^	0.918+0.068^a^
Hypersaline water (HSW; *n* = 3)	1.074+0.055^b^	0.987+0.053^a^
*P-value*	<0.05	>0.1

* Normalized Luminosity Index (mean±SEM) is expressed as a ratio of the luminosity of Alexa 488 (Nka) or Alexa 568 (Nkcc/Ncc) to DAPI (nuclei) staining within total field (0.307 mm^2^); the DAPI nuclear staining is used to correct for differences between fields.

** The data were analyzed by one-way ANOVA followed by *post-hoc* Duncan's multiple range test (*P*<0.05 is considered significant). Different alphabets indicate statistical significance from each other.

In the intestine, Nka immunoreactivity is found in the basolateral membrane of enterocytes in both the AI (**Figure 5a–5e**) and PI (**Figure 5f–5h**). Although statistically not significant, an increasing trend with salinity was observed in the normalized luminosity index for Nka in the AI and PI (**Table 1**). Nkcc/Ncc staining is strongly associated with the enterocyte brush border in both the AI (**Figure 5a–5d**) and PI (**Figure 5f–5h**). A significantly (*P*<0.05) higher normalized luminosity index for Nkcc/Ncc immunostaining was observed in the AI with increasing salinity and in the PI of HSW-acclimated fish when compared with FW-acclimated fish (**Table 1**). Cftr immunoreactivity is only consistently detected in the AI of FW fish associated with a small population of cells in the intestinal epithelium (**Figure 5e**).

## Discussion

We have quantified expression levels of seven major ion transporters (*nkcc1a*, *nkcc1b*, *nkcc2*, *ncc*, *cftr*, and *nka-α1*, and *nka-α3*) and performed immunohistochemical localization of the encoded proteins that are responsible for sodium and chloride ion regulation in gills and the intestine of tilapia acclimated to FW, SW and HSW environments. Based on the gene expression and immunohistochemical findings from the present study as well as other studies, we discuss our findings to provide perspective on their roles in regulating sodium and chloride fluxes in the gill and EGI tract under different salinity environments.

In FW environment, fish gain excess water from, and lose salt to, the low ionic and hypo-osmotic environment. With regards to iono-regulation, the fish needs to reduce and replace salt loss by actively sequestering ions from the low-salt environment via the gills and absorption from ingested food via the EGI tract. In the FW-acclimated gill, this is partly achieved with the high expression of *ncc*, *nka-α1*, and *nka-α3* as determined in this study. The Ncc located in the apical membrane sequester Na^+^/Cl^−^ from the environment to compensate for the passive losses [7], [8]. The basolaterally located Nka isoforms would transport the 3 Na^+^ outward into the plasma in exchange of 2 K^+^ inward thus generating a low intracellular Na^+^ environment that would provide a driving force for the apical Ncc to cotransport Na^+^ and Cl^−^ into the cell. The relatively low expression of *nkcc1a* and *nkcc1b* in the gill of FW-acclimated fish may correspond to some of the cells that are weakly stained with basolateral Nkcc co-localized with Nka (**Figure 4a′**).

In SW and HSW environments, fish lose water to, and gain salt from, the high ionic and hyper-osmotic environment. The SW- and HSW-acclimated fish need to replace water loss by imbibing salt water and absorbing the salt and water via the EGI tract, whereby water is retained in the body and excess salt is extruded via the gill. In both SW- and HSW-acclimated gills, our data showed that *ncc* is down-regulated markedly since apical absorption of salt is no longer needed, while *nka-α1*, *nka-α3*, *nkcc1a*, and *cftr* are up-regulated to facilitate active salt extrusion. Salt extrusion via the gill will be against an increasing electrochemical gradient hence energetically costly when fish are acclimating in SW and HSW environments. Our findings agree with the well-accepted model for active salt extrusion in SW-acclimated gills [30], [31]. The basolateral Nkcc1 is responsible for co-transporting Na^+^, K^+^ and 2 Cl^−^ from the plasma into the gill ionocytes utilizing the low intracellular Na^+^ gradient generated by Nka. The Na^+^ is pumped back into the extracellular space via the basolateral Nka while the Cl^−^ exits the cell via the apical Cftr into the external environment down its electrochemical gradient, which in turn generates a positive trans-epithelial electrical potential (TEP) that will drive the extracellular Na^+^ to exit into the external environment via leaky paracellular tight junctions between the ionocytes and neighbouring accessory cells [30]. This further explains the increase in *nka-α1*, *cftr* and *nkcc1a* in HSW-acclimated gills because the encoded proteins are required to increase active Cl^−^ excretion hence increasing TEP when salinity was increased from 30 ppt (SW) to 70 ppt (HSW). Our immunohistochemical analysis corroborated to a certain extent with the gene expression data indicating significant increases of normalized luminosity indices for Nkcc and Nka in the gills of fish-acclimated to SW and HSW when compared to FW (**Table 1**). Moreover, although not quantified, when salinity increased a noticeable increase in size and/or amount of cells expressing these proteins was apparent as shown in these representative micrographs (**Figure 4a–4c**; and **4d–4f**). As for the distinct small population of Nka immunoreactive cells which lacked Nkcc staining in gill of HSW-acclimated fish (**Figure 4c** and **4c′**), although we do not know their roles and if they are discrete cell types or merely transitional maturing mitochondrion-rich cells, it is clear to us that they became more apparent hence induced by acclimation in HSW environment. Increase in the number and size of different subtypes of ionocytes in the gills with increasing salinity had been well documented in tilapia [6]–[9].

In the EGI tract of fish, imbibed SW is first desalinated in the esophagus. The entry of Na^+^ and Cl^−^ could involve combination of Na^+^/H^+^ (Nhe) and Cl^−^/HCO_3_^−^ exchangers which are reported to be vital in esophageal salt absorption while Nkcc2 plays a major role in the EGI tract [13]. This is accompanied by the high *nka-α1* and *nka-α*3 expressions in esophagus and stomach which were significantly up-regulated in HSW-acclimated fish (**Figure 2b and 2c**) suggesting a response to salinity challenge but may be utilizing Nhe and Cl^−^/HCO_3_^−^ exchangers for their iono-regulatory roles since *nkcc2* expression was very low (**Figure 1c**). The absorption of Na^+^ and Cl^−^ in the intestine is likely facilitated by the apical Nkcc2 which is abundantly expressed in the intestinal segments (**Figure 5**). The apical Nkcc2 co-transports Na^+^, K^+^ and 2 Cl^−^ from the ingested food and fluid in the lumen into the enterocytes utilizing the low intracellular Na^+^ gradient generated by the basolateral Nka which were also abundantly expressed in the intestine (**Figure 5**). Absorption of salt from the diet via the EGI tract is essential for iono-regulation in FW-acclimated fish [32] which may explain the high expression of *nkcc2* and the encoded protein in the intestinal segments especially the PI of FW-acclimated fish detected in the present study (**Figure 1c** and **5f**). As euryhaline fish acclimate to a SW environment, its drinking rate will increase 10- to 50-fold in order to compensate for water losses [33]. Greater than 95% of the NaCl in the ingested SW is absorbed in the EGI tract which in turn helps to drive the absorption of 70–85% of the volume of ingested fluid while the remaining volume is excreted via the rectum [4], [13]. The high expression of *nkcc2* and/or the encoded protein has been reported in the EGI tract of fish acclimated to FW and SW [34]–[36]. Consistent with our findings, *nkcc2* was significantly up-regulated in the AI and MI of SW-acclimated fish compared to FW-acclimated fish reflecting its importance in the gut segments during salinity challenge. The expression of *nkcc2* in the AI, MI and PI were at comparable high levels in SW-acclimated fish, although *nkcc2* appeared down-regulated in the PI of SW-acclimated fish when compared to the very high transcript abundance in the PI of FW-acclimated fish. In HSW-acclimated fish, we observed a significant up-regulation of *nkcc2* and *nka-α1* in the respective AI, MI and PI when compared to SW-acclimated fish. This was partly corroborated with the significantly higher normalized luminosity index for Nkcc in the AI of SW- and HSW-acclimated fish, but only in PI of HSW-acclimated fish, when compared to FW-acclimated fish (**Table 1**). While the normalized luminosity index for Nka displayed an increasing trend with salinity, it was statistically not significant in the AI and PI. These findings further highlight the functional importance of the intestinal segments, especially AI and PI, in iono-regulation when salinity increases from 30 ppt to 70 ppt whereby salt load across the intestine has been estimated to quadruple [3].

The presence of a Cftr paralogue to partly facilitate basolateral exit of Cl^−^ was proposed by Grosell [13] and basolateral Cftr has been detected in *Dicentrachus labrax* during ontogeny, presumably to aid Cl^−^ exit [37]. This may explain the up-regulation of *cftr* in the intestinal segments due to the increased Cl^−^ uptake in HSW-acclimated fish. However, we could not detect basolateral Cftr using an immunohistochemical method. This may be due to the dispersed distribution of Cftr on the basolateral membrane that diffuses the fluorescent signal unlike the more focal or concentrated localization of Cftr on the apical membrane that enhances fluorescent signal detection. It may also be that the basolateral Cftr expressed in the intestine of SW- and HSW- acclimated fish is of a different isoform and is not recognizable by the monoclonal antibody raised against a small specific epitope of apical Cftr.

Intestinal salt uptake is essential to facilitate water absorption from ingested fluid to replace water loss to the external hyperosmotic environment. It has been proposed that the exit of Na^+^ and Cl^−^ into the lateral intercellular space between enterocytes may create a localized hypertonic fluid that will draw water osmotically from the luminal fluid into the plasma, in a process known as solute-linked water transport hence indirectly coupling salt uptake with water absorption [13], [38]. Another possible mechanism that coupled salt uptake with water absorption involved a more direct role of NKCC isoform which could co-transport salt together with water across membrane regardless of osmotic gradients [39]. Using primary cultures from the human corpus cilliare epithelium of the eye, it was shown that ion fluxes mediated by NKCC1 could lead to water fluxes against osmotic gradient and it was estimated about 570 water molecules are co-transported with every cycle of 1 Na^+^, 1 K^+^ and 2 Cl^−^ by NKCC1 [40]. In our present study, it is not known whether the Nkcc isoforms are indirectly [13], [38] or directly [39], [40] coupling salt and water absorption in the fish intestine. Nevertheless, our study has shown that the abundant expression of intestinal *nkcc* isoforms and their up-regulation in fish acclimated to HSW clearly underscore the necessity of salt uptake to facilitate water uptake hence further emphasizing the role of Nkcc in coupling salt and water absorption.

Since an organism will lose water to a hyperosmotic environment, the most important criterion to survive in hypersalinity is to be able to replace water loss. The ability of the Mozambique tilapia to significantly up-regulate intestinal key ion transporters, especially in the posterior intestine, when challenged with increasing extreme salinity is crucial to facilitate water uptake in order to replenish the enormous water loss in hypersaline environment. This may be one major factor that enables it to survive in extreme salinity. In hypersaline environment, the significant increase of key ion transporters in the anterior and middle intestine (as observed in our data), while important, is likely not sufficient to reduce salt content of imbibed hypersaline water and facilitate water uptake to the levels that it can replenish the enormous water loss. Therefore, the ability to up-regulate key ion transporters significantly in the posterior intestine to further facilitate water uptake becomes the determining factor that is critical for its survival in 70 ppt. This ability may not be found in other euryhaline fish. This hypothesis can further be tested using the euryhaline Nile tilapia which is unable to survive in extreme salinity and by investigating the responsiveness of the expression of the key ion transporters in the intestinal tract especially in the posterior intestine under increasing salinity in future study.

## Conclusions

We have successfully characterized the expression of key ion transporters for Na^+^ and Cl^−^ in the gill and EGI tract of the euryhaline Mozambique tilapia model acclimated to FW, SW, and HSW environments. The study provided new insights into the responsiveness of these genes and their encoded proteins along the different segments of the EGI tract of tilapia acclimated to different salinity. With respect to the genes and tissues investigated, we have identified the gill and PI as tissues most responsive to salinity challenge followed by AI and MI, thus confirming the hypothesis that different segments of the EGI tract display different levels of iono-osmoregulatory significance. Despite being an important iono-osmoregulatory organ especially in a salinity challenging environment, little is known regarding the role of the EGI tract in the Mozambique tilapia model. There is no information on the expression of the selected key ion transporter genes in different segments of the EGI tract under different salinity conditions. This communication represents the first study that provides detail absolute quantification of the expression of key ion transporter genes in the EGI tract under three different salinity conditions. The immunohistochemical staining performed in the anterior and posterior intestines in this study is entirely new and confirmed that, besides gene expression, some of the encoded proteins are indeed expressed in these tissues. More importantly, based on the gene expression profile of the *nkcc2* and *nka-α1*, we provided novel evidence that the posterior intestine, which traditionally is thought to be mainly important for water absorption, is also vital for salt absorption and iono-regulation. The high expressions of *nkcc2* and *nka-α1* in posterior intestine in FW suggest its crucial iono-regulatory role in salt absorption from food in order to replenish salt loss in the FW environment. When challenged with increasing salinity, it is a general notion that salt absorption occurs at the anterior of the EGI tract to lower the salt content of the ingested salt water in order to facilitate water absorption in the posterior intestine. However, contrary to the general notion, our study detected abundant expression of *nkcc* isoforms, *cftr* and *nka isoforms*, along the intestinal tract in response to increasing salinity challenge. This suggests that salt absorption occurs throughout the intestinal tract and becomes more intense in the posterior intestine during hypersalinity challenge as evidenced by the further up-regulation of all the investigated genes (except *ncc*) in the posterior intestine of fish in HSW when compared to SW environment. The ability of the Mozambique tilapia to significantly up-regulate intestinal key ion transporters, especially in the posterior intestine to facilitate water uptake may be one major factor that enables it to survive in extreme salinity. This study has generated novel findings, insights and ideas which represent an important milestone for the Mozambique tilapia model and paves the way for more focused studies to be done in the future with regards to the EGI tract. The expression of these genes can be used as biomarkers to further delineate the osmoregulatory role of different segments of the EGI tract for future study using the euryhaline Mozambique tilapia model.

## Supporting Information

Table S1 **Primers used for partial and full length cloning of the ion transporters used in this study.**
(PDF)

Table S2 **Primers and related information for quantitative real-time PCR used in this study.**
(PDF)
