# Supplementary material for: Expression of Key Ion Transporters in the Gill and Esophageal-Gastrointestinal Tract of Euryhaline Mozambique Tilapia Oreochromis mossambicus Acclimated to Fresh Water, Seawater and Hypersaline Water
Source: PLoS One. 2014 Jan 31;9(1):e87591. doi: 10.1371/journal.pone.0087591 (PMC3909219; doi:10.1371/journal.pone.0087591)
Supplement: Table S1 — Primers used for partial and full length cloning of the ion transporters used in this study. (PDF) [file pone.0087591.s001.pdf]

Table S1. Primers used for partial and full length cloning of the ion transporters used in this study.

Tilapia gene	GenBank accession No.	Sense (5 ′ to 3′)*	Antisense (5 ′to 3′)*	Insert size (bp)
nkcc1a	AY513737	CTAGTCTAGA**<u>ATGTCAGCACCATCCTCTG</u>**	CCGCTCGAG**<u>TTAAGAGTAGAAGGTGAGGAC</u>**	3456
nkcc1b	AY513738	CTAGTCTAGA**<u>ATGTCGGGTCAGAAACCG</u>**	CCGCTCGAG**<u>GGAGTAAAAGGTGAGGACG</u>**	3288
nkcc2	AY513739	CTAGTCTAGACC**<u>ATGGAGAGATTCAAGTCTAAC</u>**	CGGCTCGAG**<u>AGAGTAGAAAGTGAGCACG</u>**	3126
ncc	EU518934	CTAGTCTAGA**<u>ATGGGGCAGTTCAACTCTA</u>**	CCGCTCGAG**<u>CTGGCAGTAGAAAGTGAGG</u>**	3003
cftr	AB601825	CTAGTCTAGA**<u>AAGGCTTTGAACACTCACAC</u>**	CCGCTCGAG**<u>TTAGAGTCTCGTGTCATGGA</u>**	1206 (1-1206)
nka-α*1*	U82549	CTAGTCTAGA**<u>ATGGGGCTTGGGAAAGGGA</u>**	CCGCTCGAG**<u>GCCCTCTAGACTGGAAGCCAG</u>**	837 (142-978)
nka-α*3*	AF109409	CTAGTCTAGA**<u>ATGGGGGACAAAGATGACC</u>**	CCGCTCGAG**<u>CTTAGCCATACGCTTGGC</u>**	1020 (205-1224)

* The insert of the gene is underlined in bold fonts.
