# Supplementary material for: Expression of Key Ion Transporters in the Gill and Esophageal-Gastrointestinal Tract of Euryhaline Mozambique Tilapia Oreochromis mossambicus Acclimated to Fresh Water, Seawater and Hypersaline Water
Source: PLoS One. 2014 Jan 31;9(1):e87591. doi: 10.1371/journal.pone.0087591 (PMC3909219; doi:10.1371/journal.pone.0087591)
Supplement: Table S2 — Primers and related information for quantitative real-time PCR used in this study. (PDF) [file pone.0087591.s002.pdf]

Table S2. Primers and related information for quantitative real-time PCR used in this study.

Gene name	Primer name	Sequence	Amplicon size (bp)	Accession	Description
nkcc1a	NKCC1a-F	5'-GGCCACCAGCCTCCAGAACA-3'	113	AY513737	Oreochromis mossambicus Na-K-Cl cotransporter (NKCC1alpha) mRNA, complete cds
	NKCC1a-R	5'-TCAGAGGGACTGTCGGGCTCT-3'			
nkcc1b	NKCC1b-F	5'-GCTGAGAGGCTGAAGGCCCA-3'	124	AY513738	Oreochromis mossambicus Na-K-Cl cotransporter (NKCC1beta) mRNA, complete cds
	NKCC1b-R	5'-TGGCAGTGCTGGAGTGTTCCT-3'			
nkcc2	NKCC2-F	5'-GTGTCTGGGTTTGGTCCTCT-3'	127	AY513739	Oreochromis mossambicus Na-K-Cl cotransporter (NKCC2) mRNA, complete cds
	NKCC2-R	5'-GGGCCCTGTAGATGTTGTCT-3'			
ncc	NCC-F	5'-CACAAAATACCACCCTCGTG-3'	122	EU518934	Oreochromis mossambicus putative Na-Cl cotransporter (NCC) mRNA, complete cds
	NCC-R	5'-CACAAGCACTACAGGGCAGT-3'			
cftr	CFTR-F	5'-GGTGCCACAGAAAGTCTTCA-3'	139	AB601825	Oreochromis mossambicus cftr mRNA for cystic fibrosis transmembrane conductance regulator, partial cds
	CFTR-R	5'-CTTGTCTGGGAACTGCTCAA-3'			
nka-α1	NKA-a1-F	5'-CATGCTGCTGTGGATTGGTGCC-3'	129	U82549	Tilapia mossambica sodium/potassium-transporting ATPase alpha-1 subunit mRNA, complete cds
	NKA-a1-R	5'-CCGGTGATGATGACGACAGCAGA-3'			
nka-α3	NKA-a3-F	5'-GGCCGCATTGCTACTCTGACG-3'	110	AF109409	Tilapia mossambica sodium/potassium-transporting ATPase alpha-3 subunit mRNA, complete cds
	NKA-a3-R	5'-ACGCCCAAGAAGACAGCCACT-3'			
